# Cyclodextrin-Based Metal-Organic Nanotube as Fluorescent Probe for Selective *Turn-On* Detection of Hydrogen Sulfide in Living Cells Based on H_2_S-Involved Coordination Mechanism

**DOI:** 10.1038/srep21951

**Published:** 2016-02-25

**Authors:** Xuelian Xin, Jingxin Wang, Chuanfang Gong, Hai Xu, Rongming Wang, Shijie Ji, Hanxiao Dong, Qingguo Meng, Liangliang Zhang, Fangna Dai, Daofeng Sun

**Affiliations:** 1 State Key Laboratory of Heavy Oil Processing, China University of Petroleum (East China), Qingdao Shandong 266580, China; 2 Centre for Bioengineering and Biotechnology, China University of Petroleum (East China), Qingdao 266580, China; 3 Chemistry & Chemical and Environmental Engineering College, Weifang University, Weifang 261061, Shandong Province, China

## Abstract

Hydrogen sulfide (H_2_S) has been considered as the third biologically gaseous messenger (gasotransmitter) after nitric oxide (NO) and carbon monoxide (CO). Fluorescent detection of H_2_S in living cells is very important to human health because it has been found that the abnormal levels of H_2_S in human body can cause Alzheimer’s disease, cancers and diabetes. Herein, we develop a cyclodextrin-based metal-organic nanotube, **CD-MONT-2**, possessing a {Pb_14_} metallamacrocycle for efficient detection of H_2_S. **CD-MONT-2′** (the guest-free form of **CD-MONT-2**) exhibits *turn-on* detection of H_2_S with high selectivity and moderate sensitivity when the material was dissolved in DMSO solution. Significantly, **CD-MONT-2′** can act as a fluorescent *turn-on* probe for highly selective detection of H_2_S in living cells. The sensing mechanism in the present work is based on the coordination of H_2_S as the auxochromic group to the central Pb(II) ion to enhance the fluorescence intensity, which is studied for the first time.

Hydrogen sulfide (H_2_S), a colourless toxic gas with rotten egg smell, possesses double-sided nature^1^. On the one hand, H_2_S is known as a dangerous industrial pollutant for many years^2^. Because of the properties of forming explosive mixtures in the air, and causing an explosion under fire or heat, the H_2_S gas has received a growing universal attention in the aspect of safety^3^,^4^. On the other hand, along with nitric oxide (NO) and carbon monoxide (CO), the H_2_S gas has been recognized as a third gaseous transmitter gas in the human body recently^5^. *In vivo*, H_2_S is generated by endogenous enzymes^6^,^7^,^8^ (such as, cystathionine *β*-synthase (CBS), cystathionine *γ*-lyase (CSE), or 3-mercaptopyruvate sulfurtransferase (MPST)) in many organs (e.g., heart, brain, kidneys, nervous system, *etc.*) and tissues (e.g., adipose tissues, *etc*.)^9^,^10^,^11^,^12^. The abnormal levels of generated H_2_S were related to Alzheimer’s disease, cancers, diabetic complications and Down’s syndrome^13^,^14^,^15^. Hence, more and more attention has been drawn to the sensitive and selective detection of H_2_S which is chosen as a target in biological systems^16^.

In the past decade, a variety of fluorescent probes were developed for rapid detection of H_2_S. Generally, the design strategies are highly dependent on the chemical properties of the physiologically active species^17^,^18^. On the basis of current research, the sensing mechanism for the fluorescent probes of H_2_S detection can be classified into three types^19^,^20^,^21^,^22^: (i) H_2_S reductive reactions; (ii) H_2_S nucleophilic reactions and (iii) metal sulfide precipitation reactions. Most of reported results are focused on design and synthesis of organic molecules with desired functional groups to detect H_2_S based on^23^,^24^,^25^ (i) and (ii) reactions, seldom are metal-organic frameworks (MOFs) or metal-organic nanotubes (MONTs). In general, MOFs or MONTs with both high selectivity and fluorescence *turn-on* in response to H_2_S are very rare^17^,^26^,^27^.

In the previous work, we described a cyclodextrin-based Pb(II) metal-organic nanotube (**CD-MONT-2**) exhibiting temperature-dependent fluorescence and adsorption of I_2_ molecules^28^. The excellent fluorescent property of **CD-MONT-2** and the high affinity of Pb(II) to S atom prompted us to study its potential in fluorescent detection of H_2_S. Herein, we report **CD-MONT-2′** (the guest-free sample of **CD-MONT-2**) as a fluorescence *turn-on* probe for H_2_S detection. Significantly, **CD-MONT-2′** can detect H_2_S in living cells with high selectivity and moderate sensitivity. Furthermore, in the present work, a new sensing mechanism that H_2_S molecules act as auxocchromic groups to interact with the central chromophore to enhance the fluorescence emission is discovered for the first time, which is quite different from previous results.

## Results and Discussion

### Structure of CD-MONT-2

**Figure 1:**
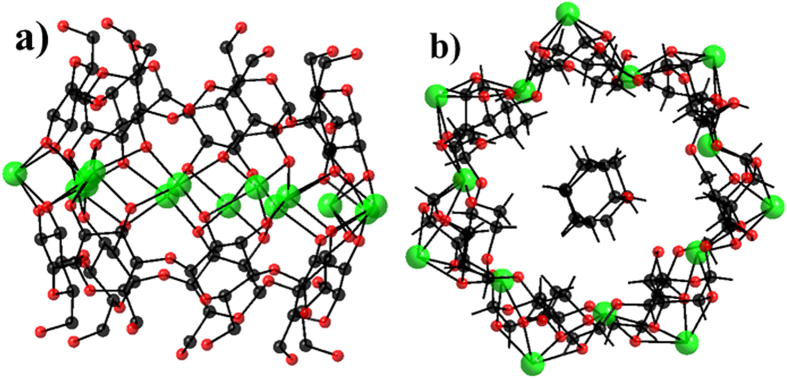
Structure of CD-MONT-2. (**a**) Side view of the structure of **CD-MONT-2** (the hydrogen atoms and cyclohexanol molecules are omitted for clarity, green: Pb(II), black: C and red: O). (**b**) Top view of the structure of **CD-MONT-2**, showing the fourteen-nuclearity lead metallamacrocycle and the uncoordinated cyclohexanol molecules in the cavity (the hydrogen atoms are omitted for clarity, green: Pb(II), black: C and red: O).

Colorless crystals of **CD-MONT-2** were obtained under the guidance of reference^28^. The cyclodextrin-based Pb(II) metal-organic nanotube (**CD-MONT-2**) consists of coplanar {Pb_14_} metallamacrocycle surrounded by two *β*-cyclodextrin molecules, as shown in Fig. 1. In the **CD-MONT-2**, the dimensions of the chiral cavity are ca. 13.0 × 10.3 × 10.2 Å filled with cyclohexanol molecules. The uncoordinated solvates in the cavity can be fully removed by heating **CD-MONT-2** at 120 °C for half an hour to generate guest-free form, **CD-MONT-2′**. The phase purity of bulk sample was further confirmed by comparison of the powder X-ray diffraction (PXRD) patterns of as-synthesized and activated sample (Supplementary Figure S1), which matched well with the simulated PXRD pattern from the single-crystal data. The following fluorescent measurements were based on **CD-MONT-2′**.

**Figure 2:**
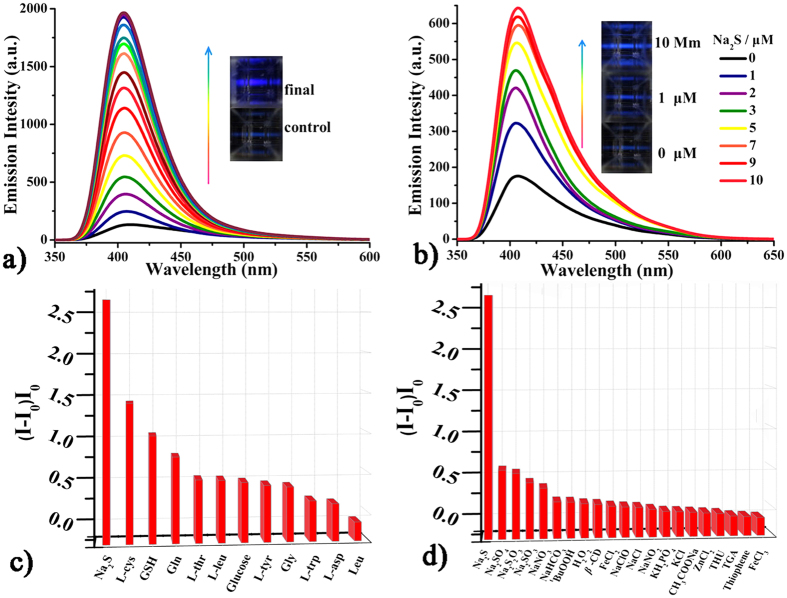
Fluorescent spectra of 10 μM CD-MONT-2′ in DMSO treated with different substances. **(a)** Addition of H_2_S after 15 min and the insert pictures were taken under Xe lamp before and finally treated with H_2_S after 15 min. **(b)** Continual addition of Na_2_S and the insert pictures were taken under Xe lamp. **(c)** Fluorescent intensity of (*I*-*I*_0_)/*I*_0_ (409 nm) spectra with 10 μM Na_2_S, 10 mM glucose and amino acids. **(d)** Fluorescent intensity of (*I*-*I*_0_)/*I*_0_ (409 nm) spectra with 10 μM Na_2_S, inorganic salts, RNS, ROS and RSS.

**Fluorescent measurements of CD-MONT-2′. CD-MONT-2′** is slightly soluble in dimethylsulphoxide (DMSO), in which **CD-MONT-2′** emits fluorescence at 409 nm (*Φ* = 0.02) upon the excitation at 330 nm (Supplementary Figure S2). To probe the fluorescent response of **CD-MONT-2′** towards H_2_S, **CD-MONT-2′** was dissolved in DMSO to make a 10 μM stock solution, then the emission spectrum was recorded from 350 to 650 nm upon the excitation at 330 nm. **CD-MONT-2′** in DMSO solution exhibits relatively weak fluorescence and keeps in the *turn*-*off* state due to the very dilute concentration. However, with the addition of H_2_S (1 mL) into the above solution, the fluorescence intensity shows a significant increase with time. Compared to the original one, almost 15 fold fluorescence enhancement is observed after 15 minutes, and no further increase occurs (Fig. 2a). The time-dependent fluorescence measurements for the addition of H_2_S into the stock solution reveal that **CD-MONT-2′** in DMSO exhibits rapid response toward H_2_S, which is different from the reported Pb-based complexes^29^. To further probe the fluorescence *turn-on* response to sulfide, various concentrations of Na_2_S (0–10 μM) were added to the stock, and the fluorescence spectra were recorded in Fig. 2b. Similarly, the fluorescence intensity clearly increases with the increasement of the concentration of Na_2_S, and almost becomes 4 times of original fluorescence intensity when the concentration of Na_2_S reaches 10 μM.

In order to confirm the fluorescent selectivity to Na_2_S over other substances, various additional experiments were carried out by gradual addition of other sodium salts (such as Na_2_SO_4_, Na_2_S_2_O_3_, Na_2_SO_3_, NaNO_3_, NaHCO_3_, NaCl, NaClO, NaOAc, FeCl_2_, FeCl_3_ and KHPO_4_), reducing agents (glucose), thiol amino acids (GSH and L-cys), non-thiol amino acids (Gln, L-Thr, L-Trp, L-Tyr, Leu, L-Leu, L-asp and Gly), reactive nitrogen species (NO_2_^−^), reactive oxygen species (H_2_O_2_ and ^t^BuOOH), and reactive sulfur species (TGA, THU and thiophene)^30^,^31^. And the spectra are shown in supplementary Figure S3–S5. The blank of only *β*-cyclodextrin in DMSO at 10 μM with Na_2_S was also recorded, and the spectrum is shown in supplementary Figure S3o. The fluorescence intensities of (*I*-*I*_0_)/*I*_0_ (where *I*_0_ is the initial fluorescence intensity, and *I* is the fluorescence intensity after the addition of the analyte) spectra (λ = 409 nm) are displayed in Fig. 2c,d. The results reveal that the additions of those substances have little effect on the fluorescence intensity of **CD-MONT-2′**, indicating the high selectivity to Na_2_S over other substances through fluorescence enhancement. All these results demonstrate that **CD-MONT-2′** exhibits fluorescence *turn*-*on* response to H_2_S molecule with high selectivity and moderate sensitivity^11^.

### Sensing mechanism

**Figure 3:**
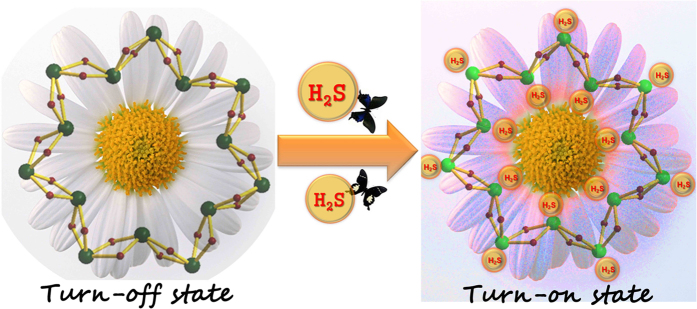
The possible mechanism for the *turn*-*on* fluorescent probe of CD-MONT-2′. The Figure is drawn by Xuelian Xin.

In the past decade, several MOF-based fluorescence *turn*-*on* probes on the detection of H_2_S were reported^19^. The reported sensing mechanism is mostly based on the H_2_S-involved organic reactions, through which the *turn-off* state of those material can be converted to *turn-on* state. Hence, there always exists a desired functional group in the MOF materials that can react with H_2_S to complete the conversion. However, in the metal-organic nanotube of **CD-MONT-2′**, there is no additional organic functional groups that can react with H_2_S to enhance the fluorescence emission. Therefore, the sensing mechanism in the present work should be different with the previous results. It is known that cyclodextrins are nonaromatic and fluorescence silent, as a result, the fluorescence emission of **CD-MONT-2′** should be assigned to a metal-centered transition involving the *s* and *p* orbitals of Pb(II) ions^28^,^32^. Thus, the fluorescence enhancement should derive from the interactions between H_2_S molecules and Pb(II) ions due to the high affinity of S atom to Pb(II) ion (*K*_sp_ of PbS: 1 × 10^−28^)^33^. As an auxochrome, the coordination of H_2_S to Pb(II) ion significantly increases the fluorescence emission of **CD-MONT-2′** (Fig. 3).

**Figure 4:**
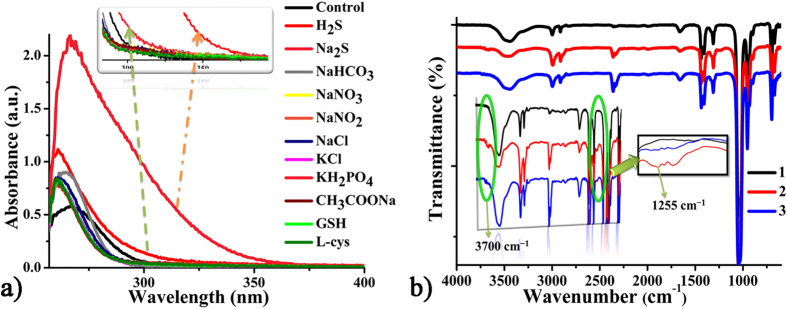
(a) UV-vis spectra of 10 μM **CD-MONT-2′** in DMSO treated with H_2_S and 10 μM different substances; (b) FTIR spectra of 10 μM **CD-MONT-2′** in DMSO with, (1) none, (2) H_2_S and (3) 10 μM Na_2_S; The insert spectra are the enlarged view of the new peaks.

To further confirm the above sensing mechanism, the UV-Vis absorbance spectra, FTIR spectra and ^1^H NMR spectra were recorded for **CD-MONT-2′** in DMSO before and after addition of H_2_S. The absorption band of **CD-MONT-2′** in DMSO appears at around 266 nm (ε = 5.68 × 10^4^ M^−1^ cm^−1^), which could be assigned to the transition from 6s^2^ to 6sp involving the lone pairs on the Pb(II)^34^. When H_2_S was added into the DMSO solution containing **CD-MONT-2′**, the absorption band enhances and shows a red-shift (Fig. 4a), which may be derived from the attachment of “S” to Pb(II)^34^. As a common auxochrome and donor, the connection of “S” could always shift the absorption to a longer wavelength and increase the absorption intensity^35^,^36^,^37^. In contrast, the addition of other substances only enhances the absorption intensity slightly and shows almost no shift. These results indicate that the sensing mechanism for **CD-MONT-2′** is based on the coordination of S atom to Pb(II) to increase the electron transfer to enhance the fluorescence intensity^38^,^39^,^40^,^41^,^42^.

Moreover, the FTIR spectra of **CD-MONT-2′** in DMSO solution before and after treated with H_2_S or Na_2_S were carried out (Fig. 4b). The absorption peaks remain unchanged, except that there is a slight difference around 1250 cm^−1^. The broad peak around 3450 cm^−1^ can be assigned to the stretching vibration of adsorbed water and hydroxyl groups in *β*-CD molecule. The peak around 1655 cm^−1^ is attributed to the O–H bending vibration of adsorbed water and hydroxyl groups in *β*-CD molecule. The absorption peak around 1033 cm^−1^ is stretching vibration of C–O–C and C–O bonds in the hole^28^,^43^,^44^,^45^. Moreover, new weak distinct peaks appeared around 1255 cm^−1^ and 3700 cm^−1^. The former peaks at about 1255 cm^−1^ can be assigned to the stretching vibration of Pb–S bond, further indicating the formation of new chemical bond (Pb–S bond)^46^,^47^,^48^; the latter peaks around 3700 cm^−1^ can be assigned to the relatively free hydroxyl group with weak hydrogen bond^49^. In addition, the ^1^H NMR spectra before and after the addition of H_2_S are shown in Supplementary Figure S6. Compared with the original one of **CD-MONT-2′**, new peaks at 7.95 ppm, 2.89 ppm and 2.73 ppm are observed in the spectra of **CD-MONT-2′** treated with H_2_S. The new peaks should be assigned to the SH which involved in the coordination or free H_2_S^36^,^50^.

### Cellular imaging experiments

**Figure 5:**
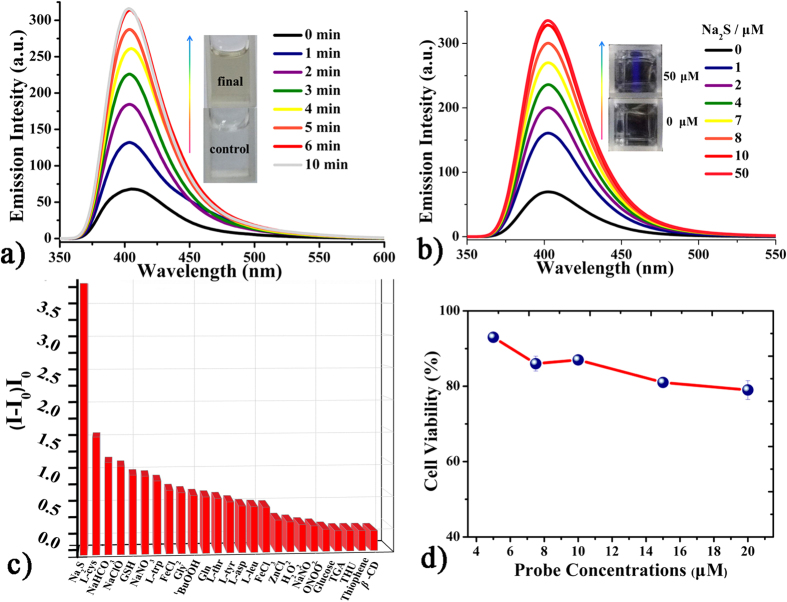
Fluorescent spectra of 0.1 μM **CD-MONT-2′** in PBS buffer (10 mM, pH 7.4, 1% DMSO) treated with, **(a)** H_2_S after 10 min, the insert pictures were taken before and finaly treated with H_2_S for 10 min. **(b)** Continual addition of Na_2_S,: the insert picture was taken in equipment under Xe lamp. **(c)** fluorescent intensity of (*I*-*I*_*0*_)/*I*_*0*_ (405 nm) spectra; **(d)** MTT assay of Hela cells in the presence of different concentrations of **CD-MONT-2′**.

The *turn-on* fluorescence sensing of H_2_S by CD-MONT-2′ prompted us to perform its potential in selective *turn-on* detection of H_2_S in living cells. To explore the fluorescent efficiency and selective response of CD-MONT-2′ towards H_2_S in the complex biological systems, CD-MONT-2′ in DMSO was diluented by PBS (phosphate buffer solution, 10 mM, pH = 7.4, the spectra are shown in Supplementary Figure S7 and the fluorescent spectra in different pH values diluented by PBS are shown in Supplementary Figure S8) at a concertation of 0.1 μM. The fluorescence measurements reveal that about 6 fold fluorescence enhancement is observed for CD-MONT-2′ in PBS buffer after 10 minutes upon the addition of H_2_S (1 mL), indicating the response toward H_2_S (Fig. 5a). Similarly, the fluorescence intensity obviously increases with the increasement of Na_2_S, and becomes almost 4 times of original fluorescence intensity when the concentration of Na_2_S reaches 10 μM (Fig. 5b, detection limit 0.058 μM, the figure is shown in Supplementary Figure S9). Moreover, in order to confirm the fluorescent selectivity, various additional experiments were carried out by gradual addition of other inorganic salts (such as NaNO_3_, NaHCO_3_, NaClO, FeCl_2_, FeCl_3_ and KHPO_4_), reducing agents (glucose), thiol amino acids (GSH and L-cys, which are known to reduce to generate *off*-*target* H_2_S detection under the action of enzymes^51^), non-thiol amino acids (Gln, L-Thr, L-Trp, L-Tyr, Leu, L-Leu, L-asp and Gly), reactive nitrogen species (NO_2_^**−**^ and ONOO^**−**^), reactive oxygen species (H_2_O_2_ and ^t^BuOOH), reactive sulfur species (TGA, THU and thiophene) into CD-MONT-2′ in DMSO diluented by PBS, and the spectra are shown in Supplementary Figure S10**–**11. The blank of only *β*-cyclodextrin in DMSO diluented by PBS at 10 μM with Na_2_S and the interference experiments were also recorded, and the spectra are shown in Supplementary Figure S10 o and Figure S12 respectively. The results demonstrate that CD-MONT-2′ exhibits fluorescence *turn*-*on* response to H_2_S molecule in the cell growth environment with high selectivity and moderate sensitivity, possessing the potential in real-time intracellular H_2_S imaging.

**Figure 6:**
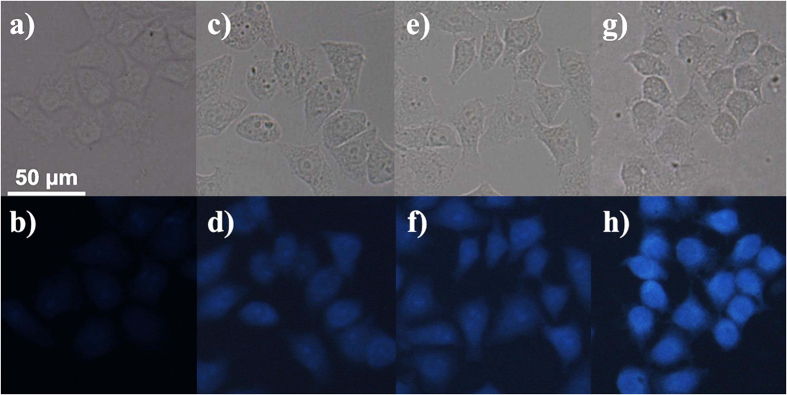
The fluorescent images of HeLa cells containing **CD-MONT-2′** incubated with increasing concentrations of Na_2_S for 15 min at 37 °C: (**b**) 0 μM, (**d**) 1 μM, (**f**) 50 μM, and (**h**) 100 μM; (**a,c,e,g**) are the bright field images of (**b,d,f,h**).

Hence, the **CD-MONT-2′** may be utilized to living cell imaging to sulphide. To test the viability and proliferation of the living cell, the MTT assay on HeLa cells was performed^50^ (Fig. 5d). The cell viability is not lower than 80% until the concentration of **CD-MONT-2′** reaches 20 μM, indicating the low toxicity at the concentration of 0.1 μM. The HeLa cells were incubated with 10 μM probe for 15 minutes at 37 °C in a 5% CO_2_ atmosphere, and washed with PBS for three times to remove the residual probe. Then fresh PBS containing various concentrations of Na_2_S were respectively added into the treated HeLa cells and incubated for 15 minutes. The fluorescent image of control one shows that **CD-MONT-2′** probe could enter inside the cell and result in the weak blue fluorescent signal. However, with the increase of sulphide (Na_2_S) concentration from 1 to 100 μM, the signal intensity increases obviously (Fig. 6). The strong blue fluorescent signal is observed when the sulphide concentration reaches 100 μM. These results confirm that **CD-MONT-2′** is active as a probe for sulphide and can be applied in living cell imaging.

**Figure 7:**
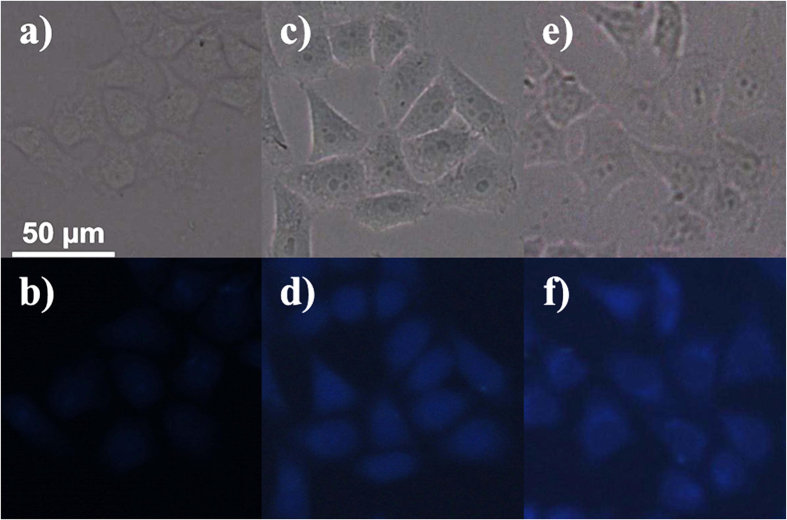
The fluorescent images of HeLa cells containing **CD-MONT-2′** incubated with the concentrations of (**b**) 0 μM, (**d**) 200 μM GSH and (**f**) 200 μM L-cys after 15 min at 37 °C; (**a,c,e**) are their bright field images of (**b,d,f**), respectively.

In addition to supplementing cells with extraneous sources of sulphide, our experiments further focus on biothiols, such as the amino acid glutathione (GSH) and L-cysteine (L-cys), which can act as potential sulphide sources^51^,^52^,^53^,^54^,^55^. After 15 minutes of incubation, addition of both thiol species (200 μM GSH or L-cys in PBS) elicits a brighter fluorescent response (see Fig. 7). The significant responses indicate that the **CD-MONT-2′** probe could detect not only external sulphides supplemented to the cell cultures, but also sulphides produced by the cells *in vivo*.

## Discussion

The design and synthesis of fluorescent *turn*-*on* probes for rapid detection of H_2_S in living cells is an active field in material chemistry and cell biology^9^,^17^. The development of coordination chemistry in the past decades opened a new avenue in searching fluorescent materials for selective detection of H_2_S. Actually, most of fluorescent coordination complexes including metal-organic frameworks show *turn*-*off* response towards H_2_S^29^, functional coordination complex-based probes with fluorescent *turn*-*on* response towards H_2_S are quite rare. Up to date, several MOF-based fluorescent *turn*-*on* probes have been synthesized and applied in the detection of H_2_S based on reduction/precipitation mechanism^19^. In the present work, the sensing mechanism is based on the coordination of H_2_S (as auxochromic group) to Pb(II) ion to enhance the fluorescent emission. To the best of our knowledge, this is the first fluorescence *turn*-*on* probe that can selectively detect H_2_S in living cells based on H_2_S-involved coordination mechanism.

On the other hand, one of the significant bottlenecks in detection of H_2_S in living cells is the toxicity of the fluorescent probe. Most of organic ligands used in the assembly of coordination complexes or metal-organic frameworks are limited to non-renewable petrochemical feedstocks and somewhat toxic. Recently, Stoddart and co-workers reported a series of MOFs composed of an edible natural product, *γ*-cyclodextrin^56^,^57^,^58^,^59^. In our work, **CD-MONT-2′** was assembled by use of *β*-cyclodextrin, which is non-toxic and increases its practical application in the fluorescent detection of H_2_S in living cells.

## Conclusions

In conclusion, a fluorescent metal-organic nanotube based on *β*-cyclodextrin for the detection of H_2_S has been developed and described. The newly developed fluorescent probe can detect H_2_S through fluorescence *turn*-*on* fashion with high selectivity and moderate sensitivity. Furthermore, the sensing mechanism is based on the coordination of H_2_S to the central metal ions of the probe to tune the fluorescence intensity, which is quite different from the results reported previously. Significantly, the use of nontoxic *β*-cyclodextrin ligand in the probe makes it more advantage in the practical application. Our study may provide a new way in design and synthesis of new functional material on fluorescence *turn*-*on* detection of H_2_S in living cells.

## Methods

### Materials and Physical Measurements

#### Materials

All chemicals and solvents were purchased and used as received without further purification. Water used in living cell experiments were processed with a Millipore Milli-Q system (18.2 M Ω·cm). Thioglycollic cid (TGA) and thiourea (THU) were purchased. ^t^BuOOH could also be used to induce ROS in biological systems^31^. The ONOO^−^ source was generated by the reaction of H_2_O_2_, H_2_SO_4_, NaNO_2_ and MnO_2_. The concentration is obtained by UV-Vis at 302 nm^60^.

#### Physical Measurements

Fluorescence spectra were recorded with a Hitachi F-7000 fluorescence spectrophotometer. The powder X-ray diffraction data were obtained on a Philips X’ Pert with Cu-Kα radiation (λ = 0.15418 nm). FTIR spectra were collected on a Bruker VERTEX-70 spectrometer in the 4000 − 600 cm^−1^ region. The optical absorption spectra were measured on a UV-vis spectrometer (Specord 205, Analytik Jena) in the range of 200 to 600 nm. ^1^H NMR spectra were recorded on a Bruker AVANCE-400 NMR Spectrometer in *d*_6_-DMSO.

### Synthesis of CD-MONT-2

*β*-CD (0.10 mmol, 115 mg) and PbCl_2_ (0.80 mmol, 225 mg) were suspended in distilled water (30 mL) and stirred at 80 °C for an hour. After cooled to room temperature, the precipitate was separated from the mixture. The obtained solution was transformed to five 6 mL of glass tubes, then 3 mL cyclohexanol and trimethylamine were layered onto the solution in each tube. The glass tubes were sealed and heated at 110 °C for 3 days. A lot of colourless rod-like crystals were collected by filtration, washed with distilled water and dried in air (yield: 78%).

### Fluorescent experiments

All fluorescent measurements were carried out at room temperature on a Hitachi F7000 fluorescence spectrophotometer. Samples were excited at 330 nm with the excitation and emission slit widths set at 20 and 10 nm, respectively. The emission spectrum was scanned from 350 to 650 nm with 1200 nm min^−1^. The photomultiplier voltage was set at 400 V. Accordingly, the probe was dissolved in dimethylsulphoxide (DMSO) to make a 10 μM stock solution and the added substances were dissolved in DMSO as well. The stock was diluented by PBS for 100 times to obtain the concentration of 0.1 μM and the added substances were dissolved in PBS. The H_2_S was made by the reaction of FeS and H_2_SO_4_ and collected in the 250 mL flask for more than 30 min. To test the time-dependent properties, 1 mL H_2_S gas was taken out from the flask and bubbled into 1 mL corresponding solution.

### MTT Cytotoxicity assay

HeLa cells were grown up in DMEM media with 10% FBS and penicillin/streptomycin. Cells were allowed to grow to 80% confluency before being collected using trypsin. Cells were transferred into a 96-well plate (Corning), and then incubated overnight at 37 °C in a 5% CO_2_ atmosphere. A serial dilution on **CD-MONT-2′** was performed in DMEM media, with 10 μL added to each well to give final concentrations of 5, 7.5, 10, 15 and 20 μM probe. Cells were allowed to incubate for 24 h. Wells containing only cells and only DMSO were also set up to serve as positive and negative controls. To test cell viability and proliferation, the MTT assay was performed. Briefly, after incubation for the indicated times, 10 μL of MTT solution (5 mg/mL) was added to each well, and the cells were incubated for further 4 h at 37 °C. The precipitated formazan was dissolved in 150 μL of dimethyl sulfoxide. The absorbance at 490 nm (A490) was measured using a microplate autoreader (Molecular Devices, M2e). Note that the wells without cells acted as the blank during the A490 measurement.

### Cellular imaging experiments

HeLa cells were grown as previously described. The cells were seeded onto 12 mm sterile coverslips in a 24-well plate (Coring) and allowed to grow to 80% confluency at 37 °C in a 5% CO_2_ atmosphere. At this time, a final concentration of 0.1 μM **CD-MONT-2**′ was added to the cells and incubated for 15 minutes at the previous conditions. Media was then removed, and PBS was added to remove the probe left in solution and optimize the background signal. The sulphur source was then added (Na_2_S, GSH, or L-cys) to the desired concentration and cells were incubated for 10–15 minutes at room temperature before imaging. And then, the cells were fixed for 20 min in 200 μL 4% paraformaldehyde (the fluorescent images of HeLa cells without being fixed are showed in Figure S13). After fix-ation, the cells were washed thrice with PBS. The coverslip with fixed cells was topped by a glass slide with a drop of 10 μL of glycerol/PBS (v/v = 1:1) and placed above the objective on a fluorescence microscope.

All imaging experiments were performed on a Leica DMI3000B Inverted fluorescence microscopic. Excitation and emission were monitored using blue fiter provided with the scope. Imaging was performed with the × 20 dry objectives which are provided with the scope. Images were captured using Leica Application Suite software.

## Additional Information

**How to cite this article**: Xin, X. *et al.* Cyclodextrin-Based Metal-Organic Nanotube as Fluorescent Probe for Selective *Turn-On* Detection of Hydrogen Sulfide in Living Cells Based on H2S-Involved Coordination Mechanism. *Sci. Rep.* **6**, 21951; doi: 10.1038/srep21951 (2016).

## Supplementary Material

Supplementary Information
