# Supplementary material for: Cyclodextrin-Based Metal-Organic Nanotube as Fluorescent Probe for Selective Turn-On Detection of Hydrogen Sulfide in Living Cells Based on H2S-Involved Coordination Mechanism
Source: Sci Rep. 2016 Feb 25;6:21951. doi: 10.1038/srep21951 (PMC4766401; doi:10.1038/srep21951)
Supplement: Supplementary Information [file srep21951-s1.doc]

Supporting Information for

**Cyclodextrin-Based Metal-Organic Nanotube as Fluorescent Probe for Selective *Turn-On* Detection of Hydrogen Sulfide in Living Cells Based on H_2_S-Involved Coordination Mechanism**

Xuelian Xin^1^, Jingxin Wang^2^, Chuanfang Gong^1^, Hai Xu^2^, Rongming Wang^1^, Shijie Ji^1^, Hanxiao Dong^1^, Qingguo Meng^3^, Liangliang Zhang^1^, Fangna Dai^1^, Daofeng Sun^1^,*

^1^ State Key Laboratory of Heavy Oil Processing, China University of Petroleum (East China), Qingdao Shandong 266580, China. ^2^ Centre for Bioengineering and Biotechnology, China University of Petroleum (East China), Qingdao 266580, China. ^3^ Chemistry & Chemical and Environmental Engineering College, Weifang University, Weifang 261061, Shandong Province, China.

Email: dfsun@upc.edu.cn

1. **PXRD of CD-MONT-2**


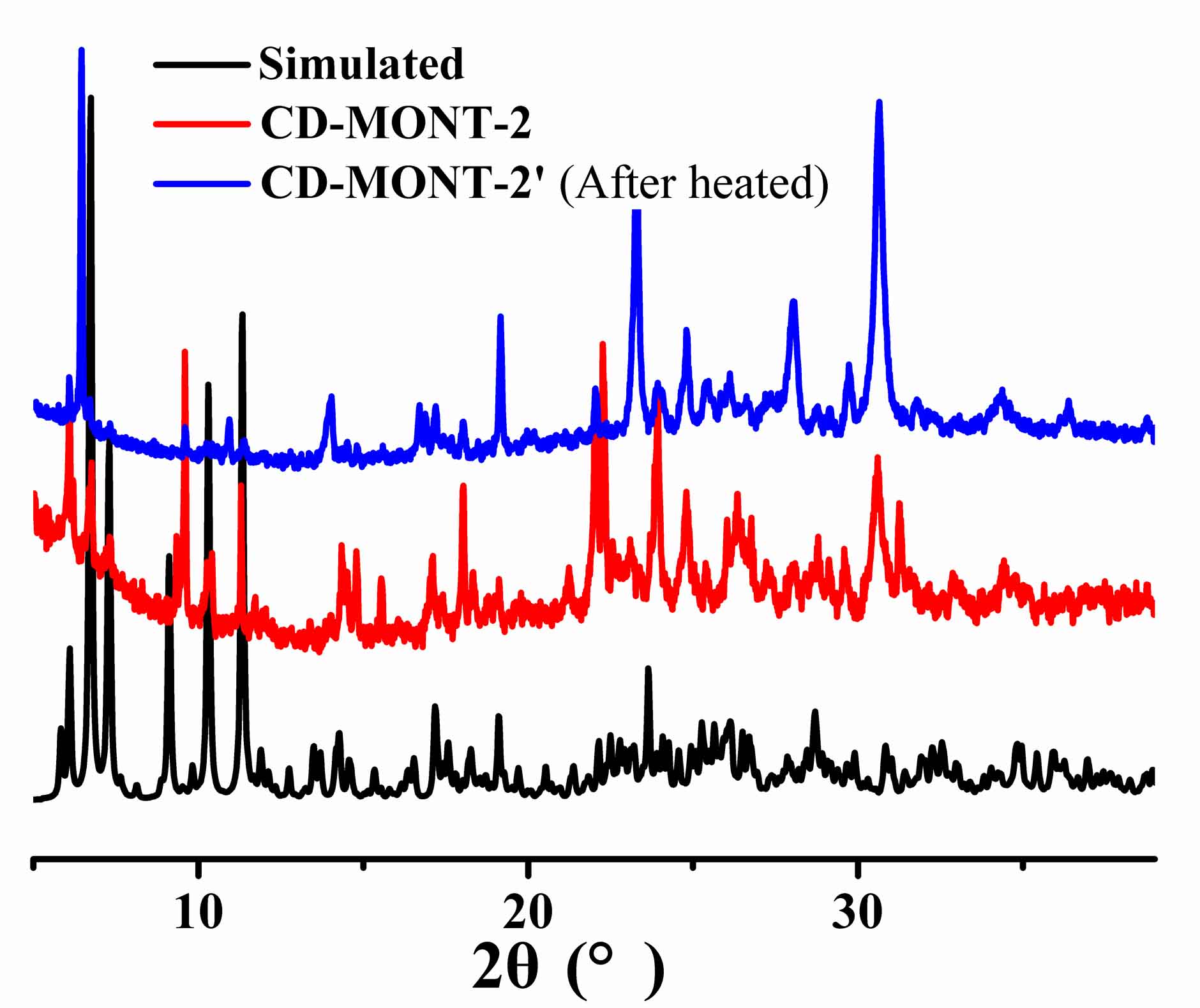


**Supplementary Figure S1 |** PXRD patterns of simulated based on crystal data, as-synthesized and after heated at 120 °C for half an hour.

1. **Fluorescent spectra of the 10 μM CD-MONT-2’ in DMSO**

**
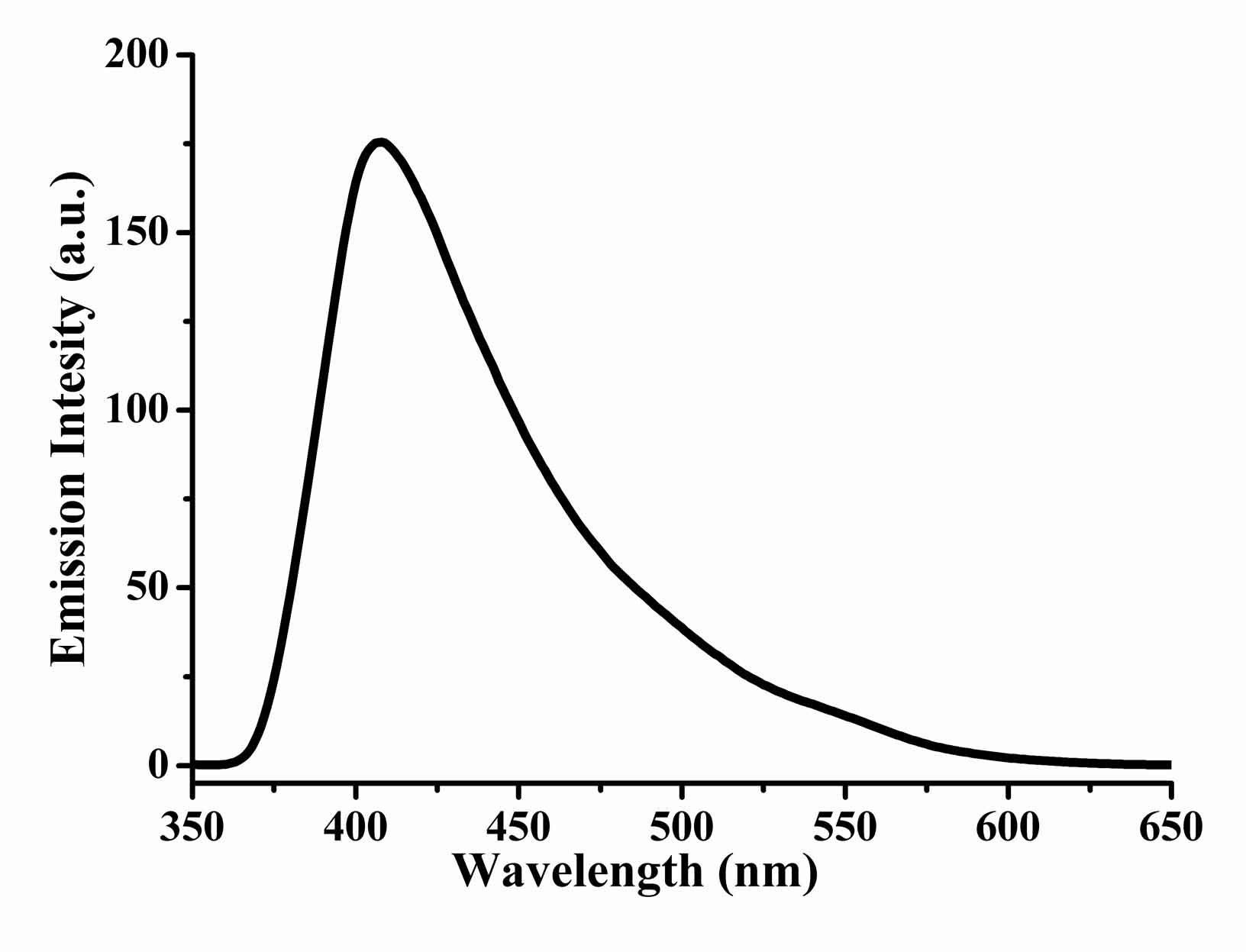
**

**Supplementary Figure S2 |** Fluorescent spectra of 10 μM **CD-MONT-2’** in DMSO.

1. **Fluorescent spectra of the CD-MONT-2’ in DMSO with different inorganic salts and *β*-cyclodextrin in DMSO at 10 μM with Na_2_S.**


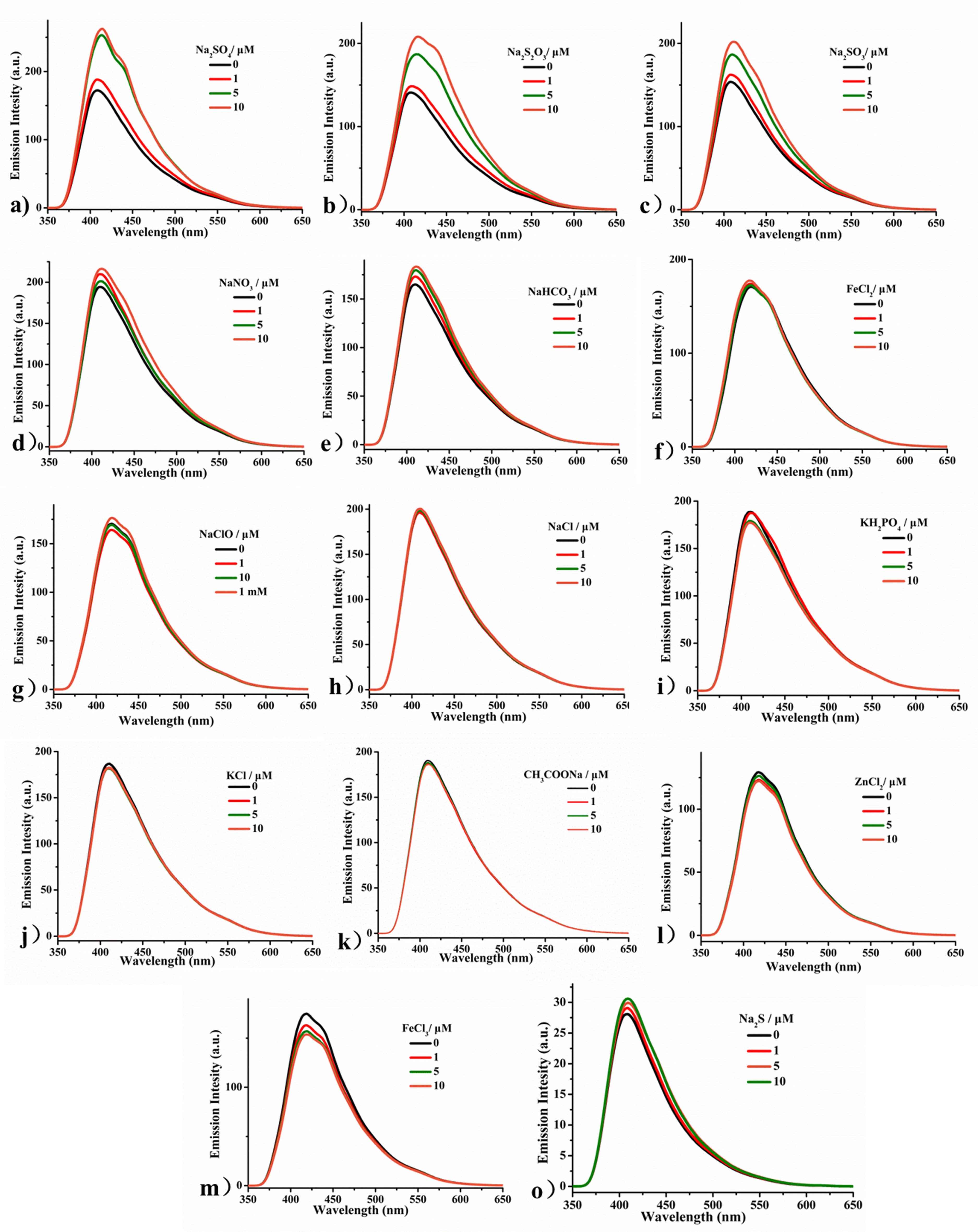


**Supplementary Figure S3 |** Fluorescent spectra of 10 μM **CD-MONT-2’** in DMSO treated with: a) Na_2_SO_4_, b) Na_2_S_2_O_3_, c) Na_2_SO_3_, d) NaNO_2_, e) NaHCO_3_, f) FeCl_2_, g), NaClO, h) NaCl, i) KH_2_PO_4_, j) KCl, k)CH_3_COONa, l) ZnCl_2_ and m) FeCl_3_ at concentrations: 0 μM, 1 μM, 5 μM and 10 μM. j) Fluorescent spectra of 10 μM *β*-CD in DMSO treated with different concentrations of Na_2_S: 0 μM, 1 μM, 5 μM and 10 μM.

1. **Fluorescent spectra of the CD-MONT-2’ in DMSO with reducing agents, thiol amino acids and non-thiol amino acids.**

**
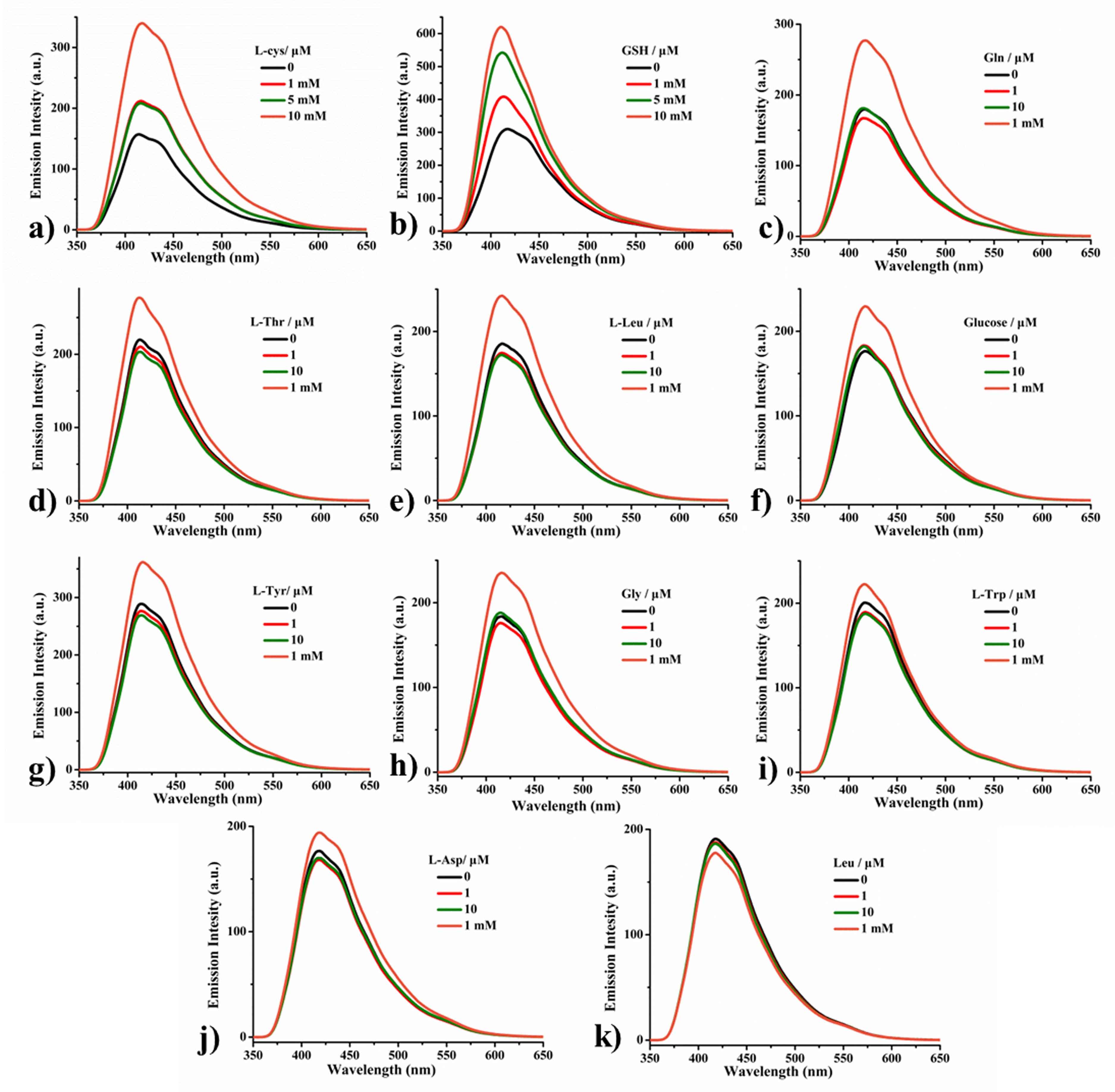
**

**Supplementary Figure S4 |** Fluorescent spectra of 10 μM **CD-MONT-2’** in DMSO treated with: a) L-cys and b) GSH at concentrations: 0 mM, 1 mM, 5 mM and 10 mM, c) Gln, d) L-thr, e) L-leu, f) glucose, g) L-tyr, h) Gly, i) L-trp, j) L-asp and k) Leu at concentrations: 0 μM, 1 μM, 10 μM, and 1 mM.

1. **Fluorescent spectra of the CD-MONT-2’ in DMSO with RNS, ROS and RSS.**


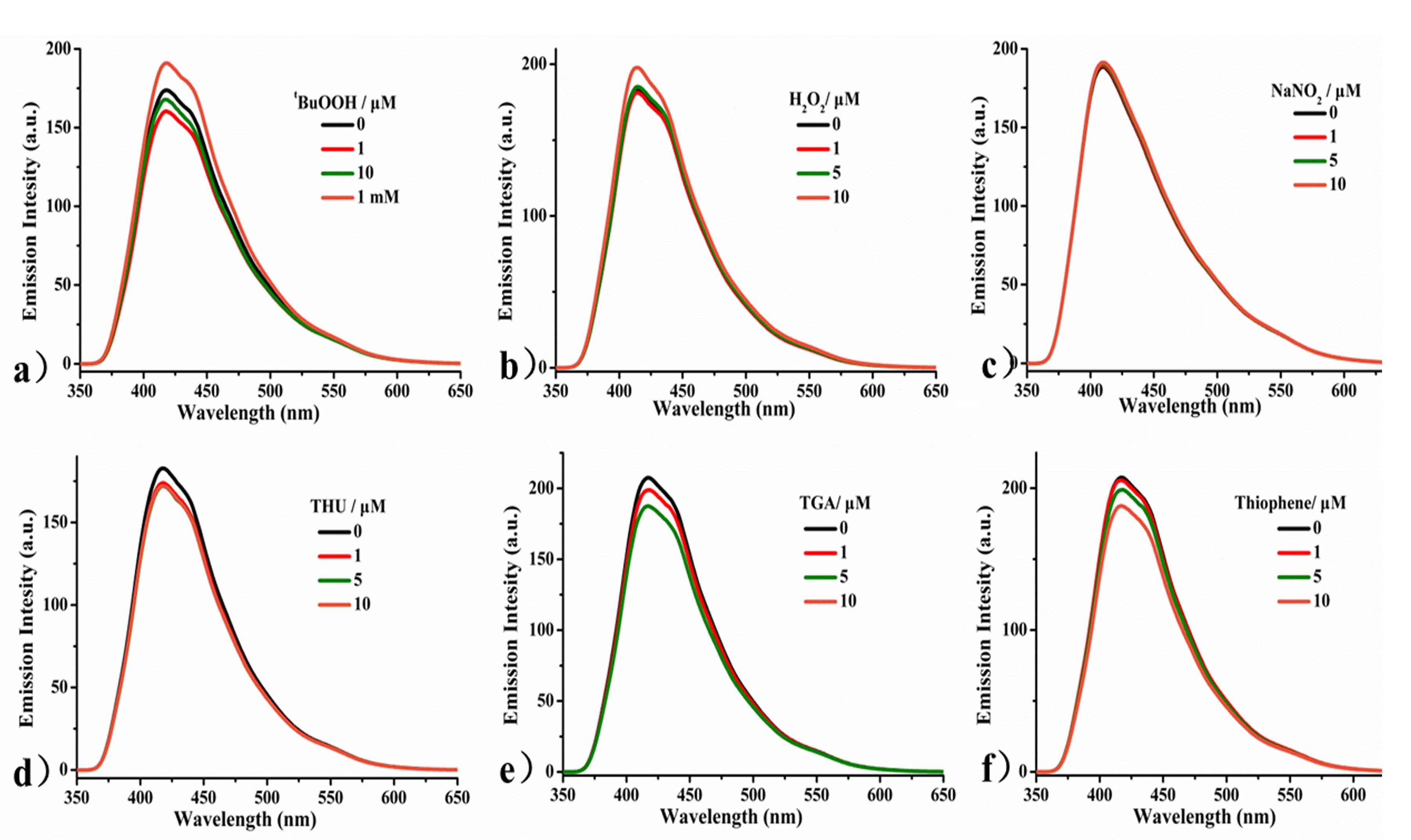


**Supplementary Figure S5 |** Fluorescent spectra of 10 μM **CD-MONT-2’** in DMSO treated with: a) ^t^BuOOH (10 mM), b) H_2_O_2_, c) NaNO_2_, d) THU, e) TGA and f) Thiophene at concentrations: 0 μM, 1 μM, 5 μM and 10 μM.

1. **^1^H NMR spectra.**

**
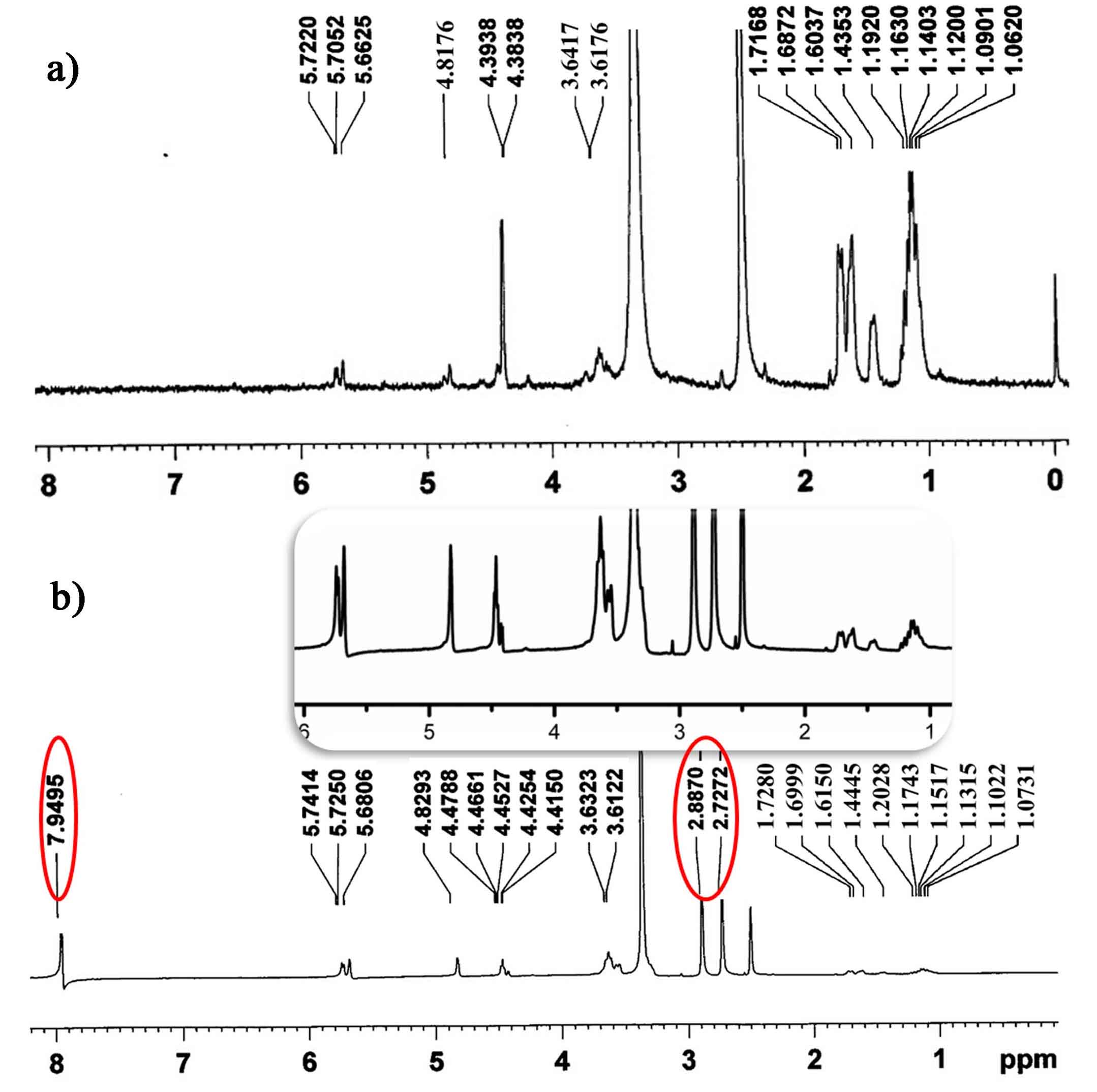
**

**Supplementary Figure S6 |** ^1^H NMR spectra of **(a)** **CD-MONT-2’** in *d*_6_-DMSO and **(b)** **CD-MONT-2’** in *d*_6_-DMSO treated with H_2_S. Insert: partial enlarged view of (b).

1. **Fluorescent spectra of the CD-MONT-2’ in DMSO diluented by PBS.**

**
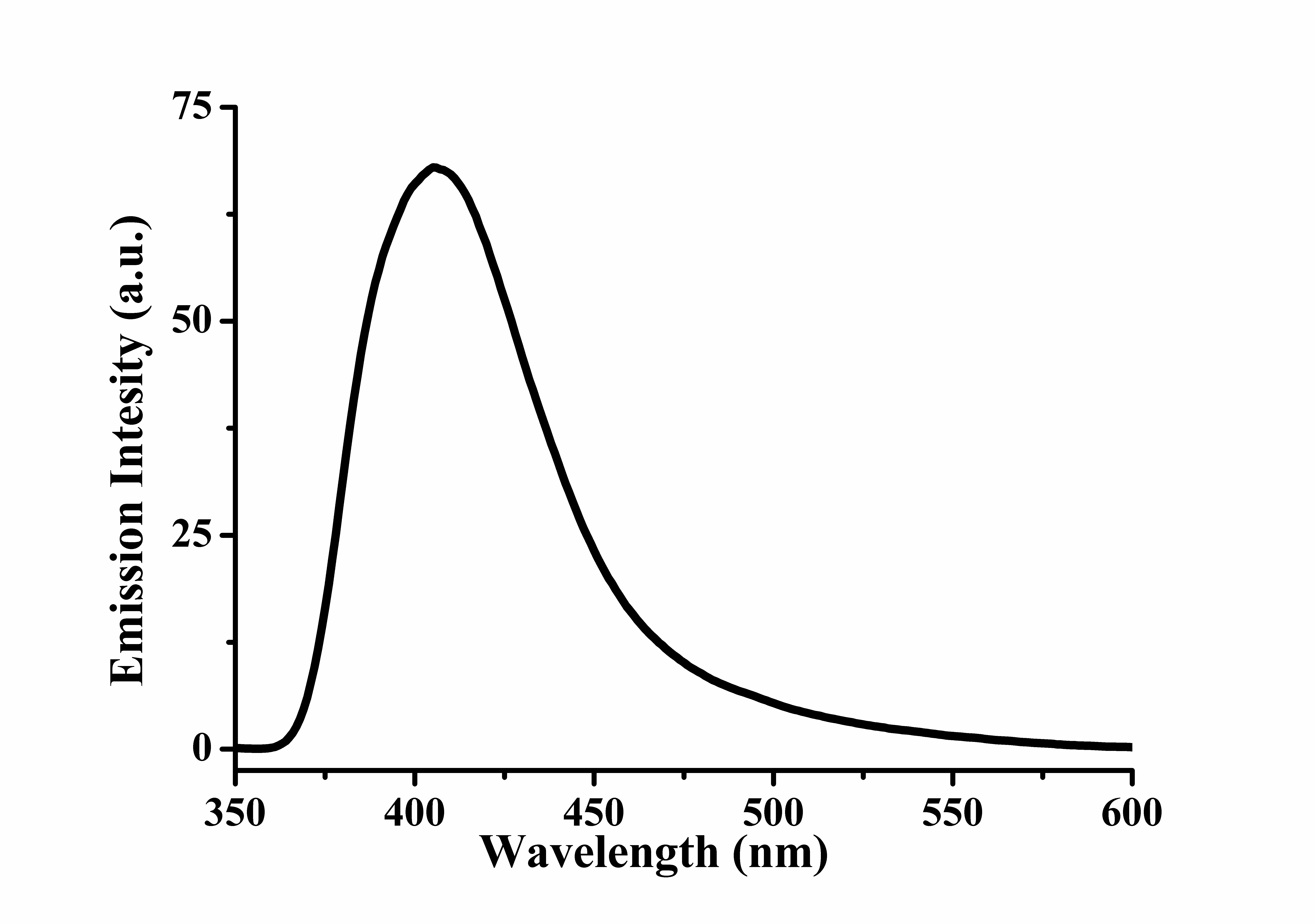
**

**Supplementary Figure S7 |** Fluorescent spectra of 0.1 μM **CD-MONT-2’** in DMSO diluented by PBS.

1. **Fluorescent spectra of 0.1 μM CD-MONT-2’ in different pH values diluented by PBS.**

**
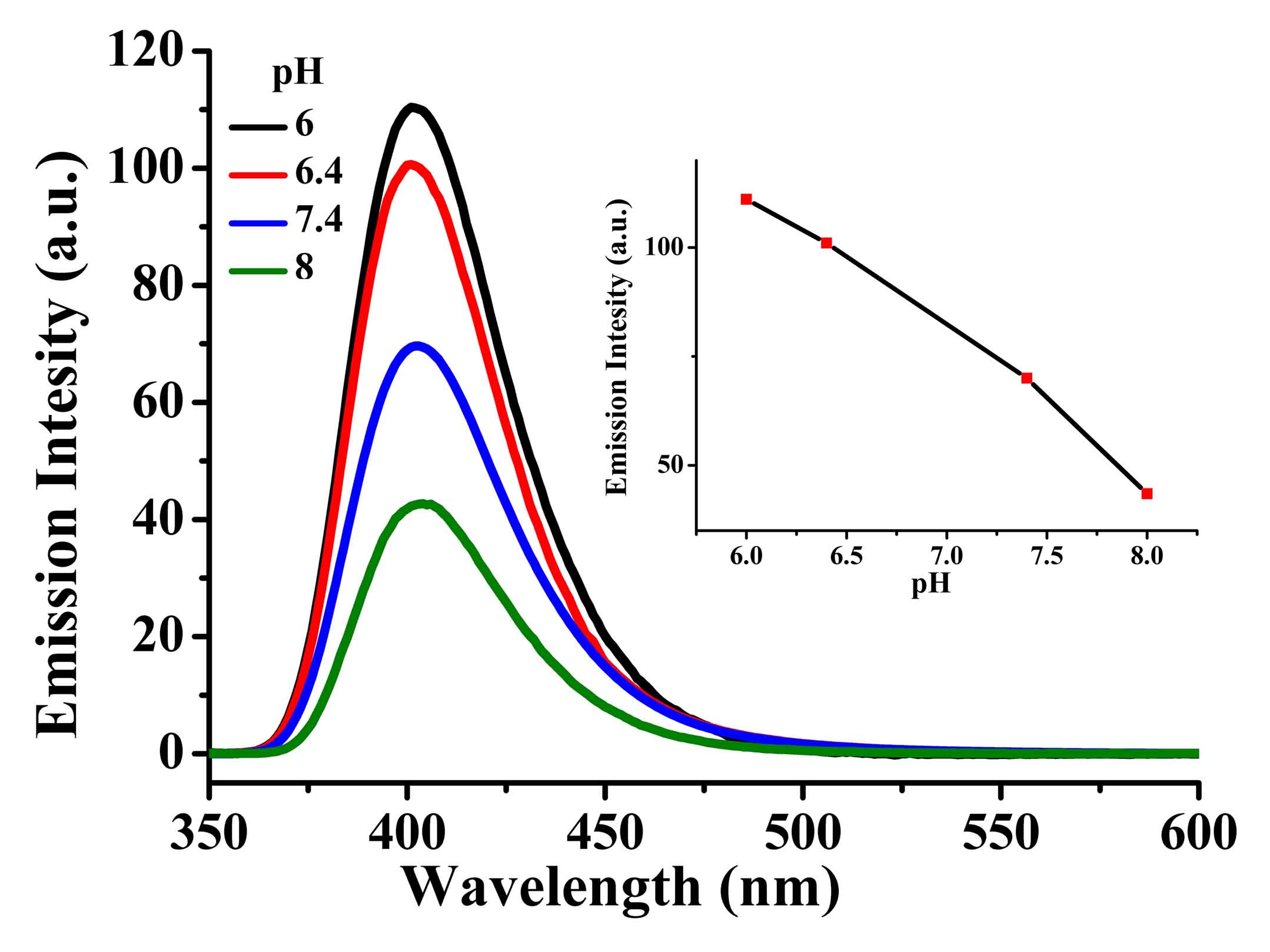
**

**Supplementary Figure S8 |** Fluorescent spectra of 0.1 μM **CD-MONT-2’** in different pH values diluented by PBS.

1. **Detection limit for H_2_S from probe.**

**
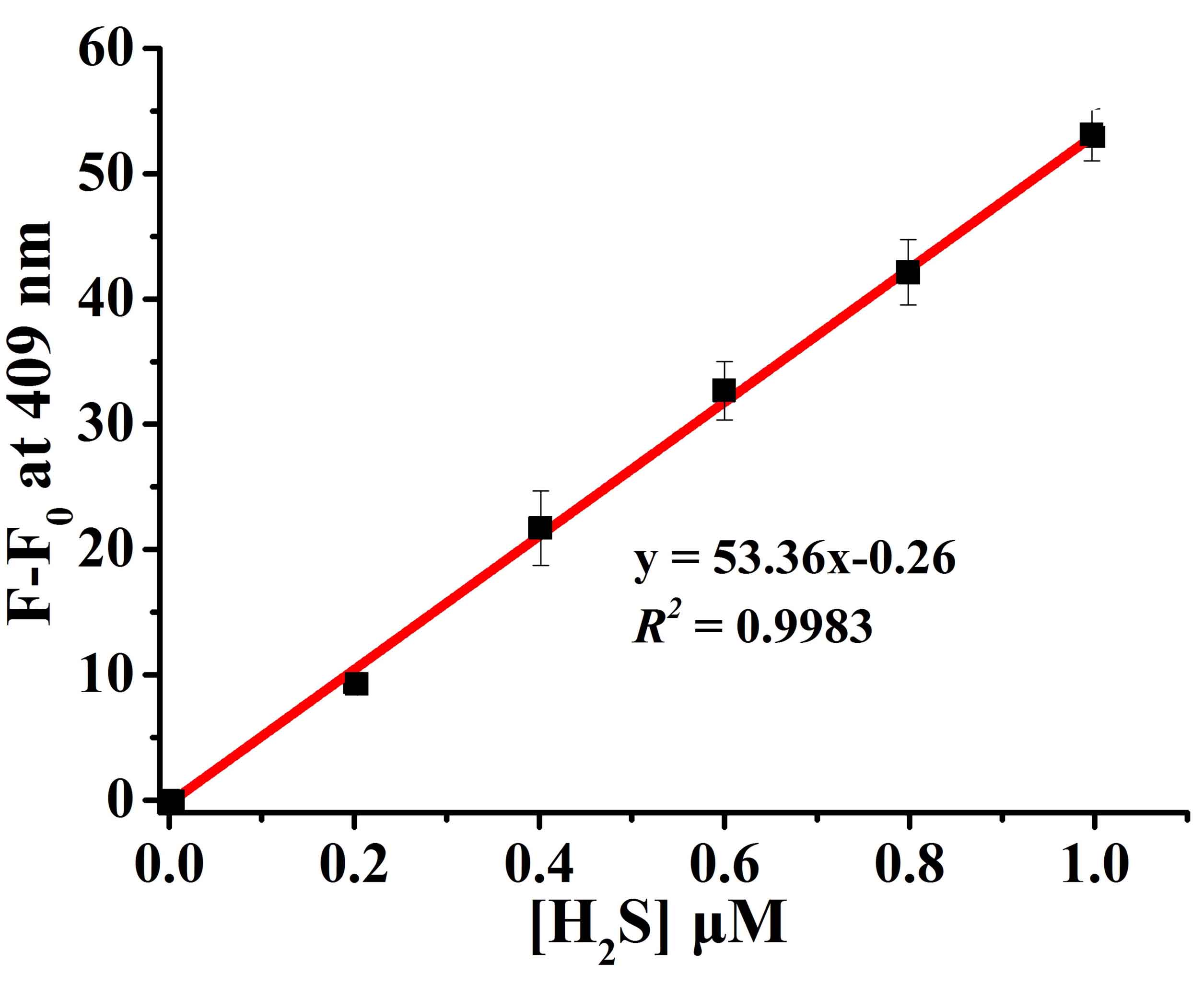
**

**Supplementary Figure S9 |** Detection limit for H_2_S from probe.

1. **Fluorescent spectra of the CD-MONT-2’ in DMSO diluented by PBS treated with different substances and *β*-cyclodextrin in diluented solution treated with Na_2_S**

**
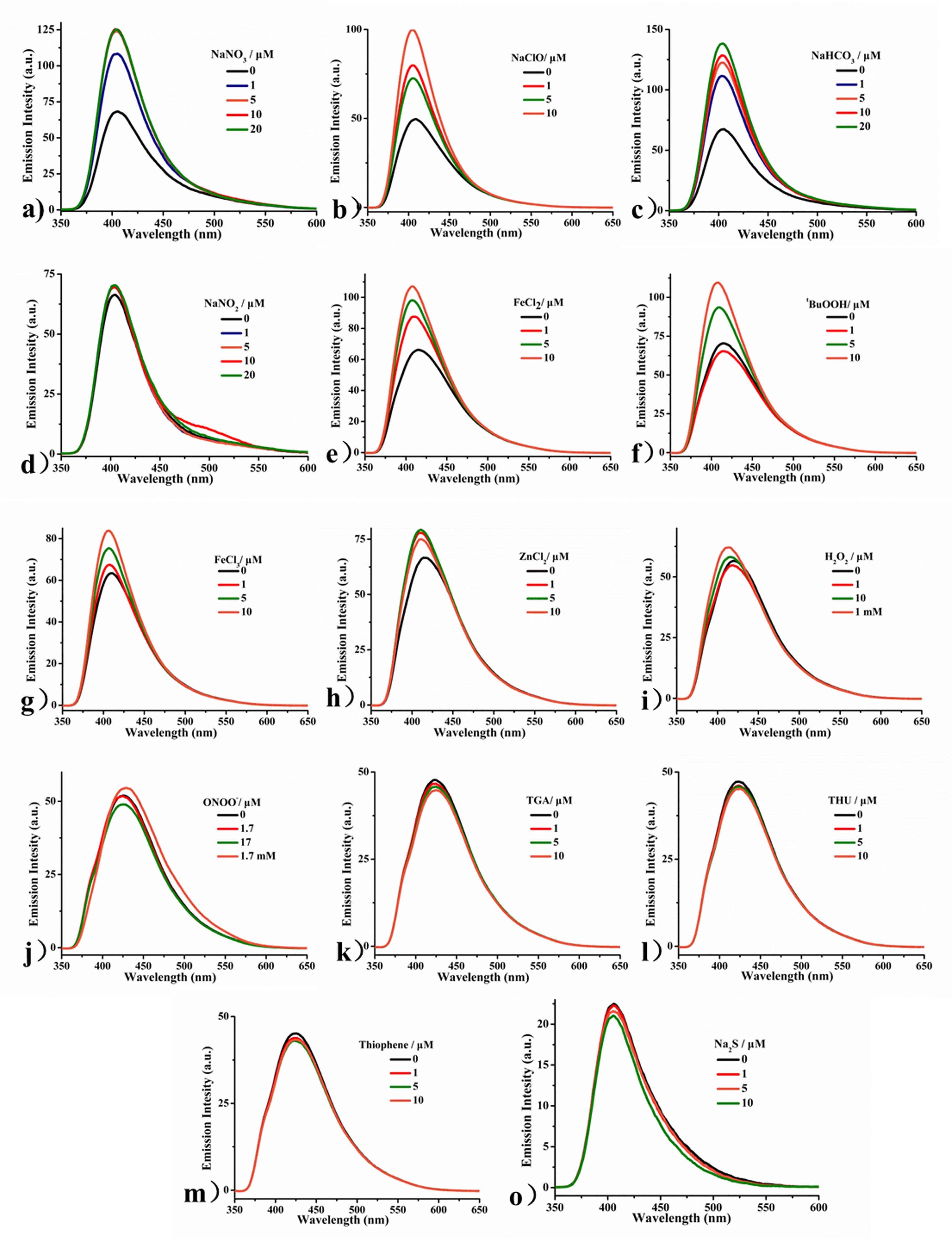
**

**Supplementary Figure S10 |** Fluorescent spectra of 0.1 μM **CD-MONT-2’** in PBS buffer (10 mM, pH 7.4, 1% DMSO) treated with: a) NaNO_3_, b) NaClO, c) NaHCO_3_, d) NaNO_2_, e) FeCl_2_, f) ^t^BuOOH, g) FeCl_3_, h) ZnCl_2_, i) H_2_O_2_, j) ONOO^−^, k) TGA, l) THU and m) Thiophene in concentrations: 0 μM, 1 μM, 5 μM and 10 μM. o) 0.1 μM *β*-CD in PBS buffer (10 mM, pH 7.4, 1% DMSO) treated with different concentrations of Na_2_S: 0 μM, 1 μM, 5 μM and 10 μM.

1. **Fluorescent spectra of the CD-MONT-2’ in DMSO diluented by PBS treated with different amino acids.**


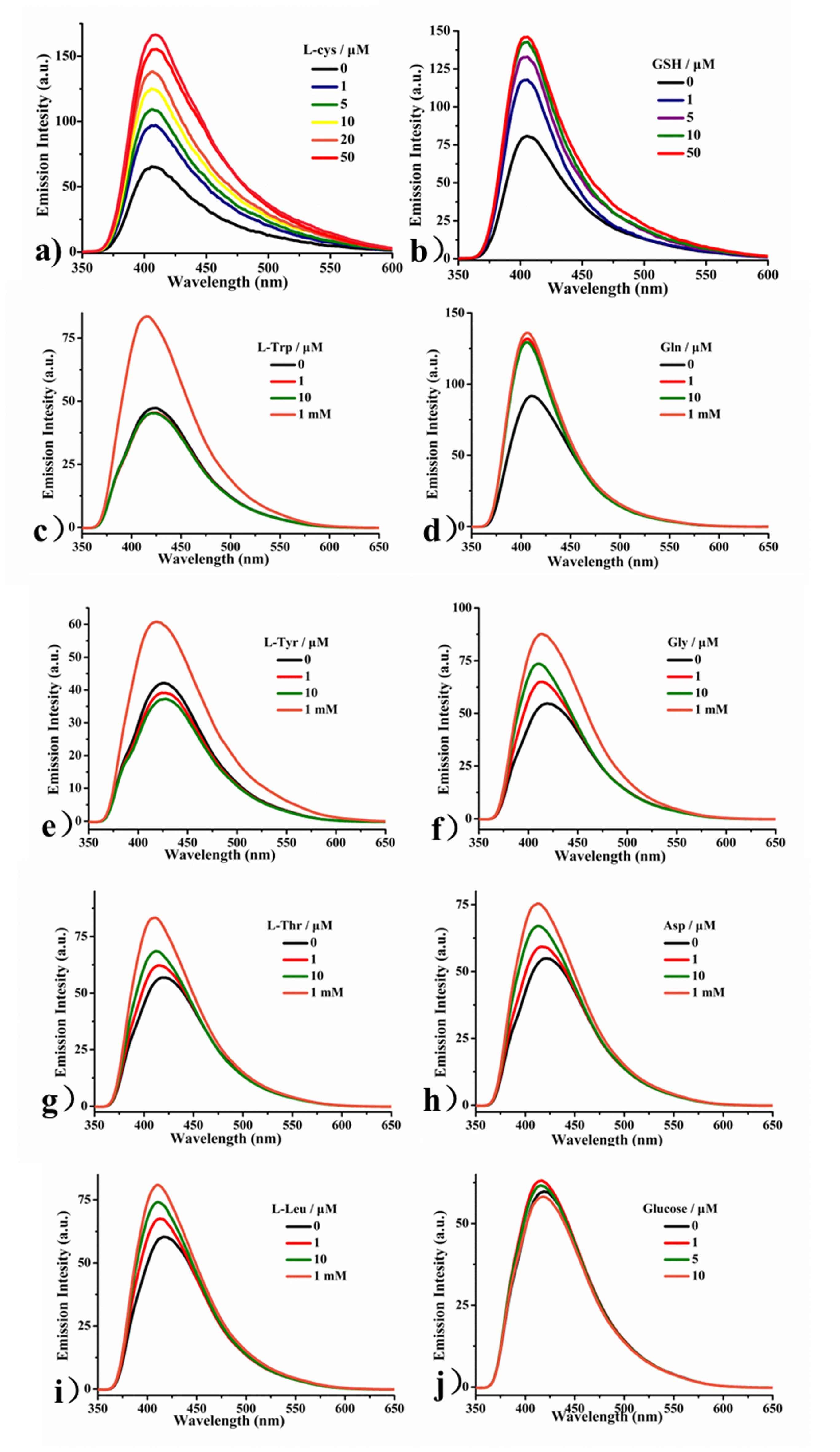


**Supplementary Figure S11 |** Fluorescent spectra of 0.1 μM **CD-MONT-2’** in PBS buffer (10 mM, pH 7.4, 1% DMSO) treated with: a) L-cys, b) GSH, c) L-trp, d) Gln, e) L-tyr, f) Gly, g) L-thr, h) L-asp, i) L-leu and j) Glucose.

1. **Interference experiments.**


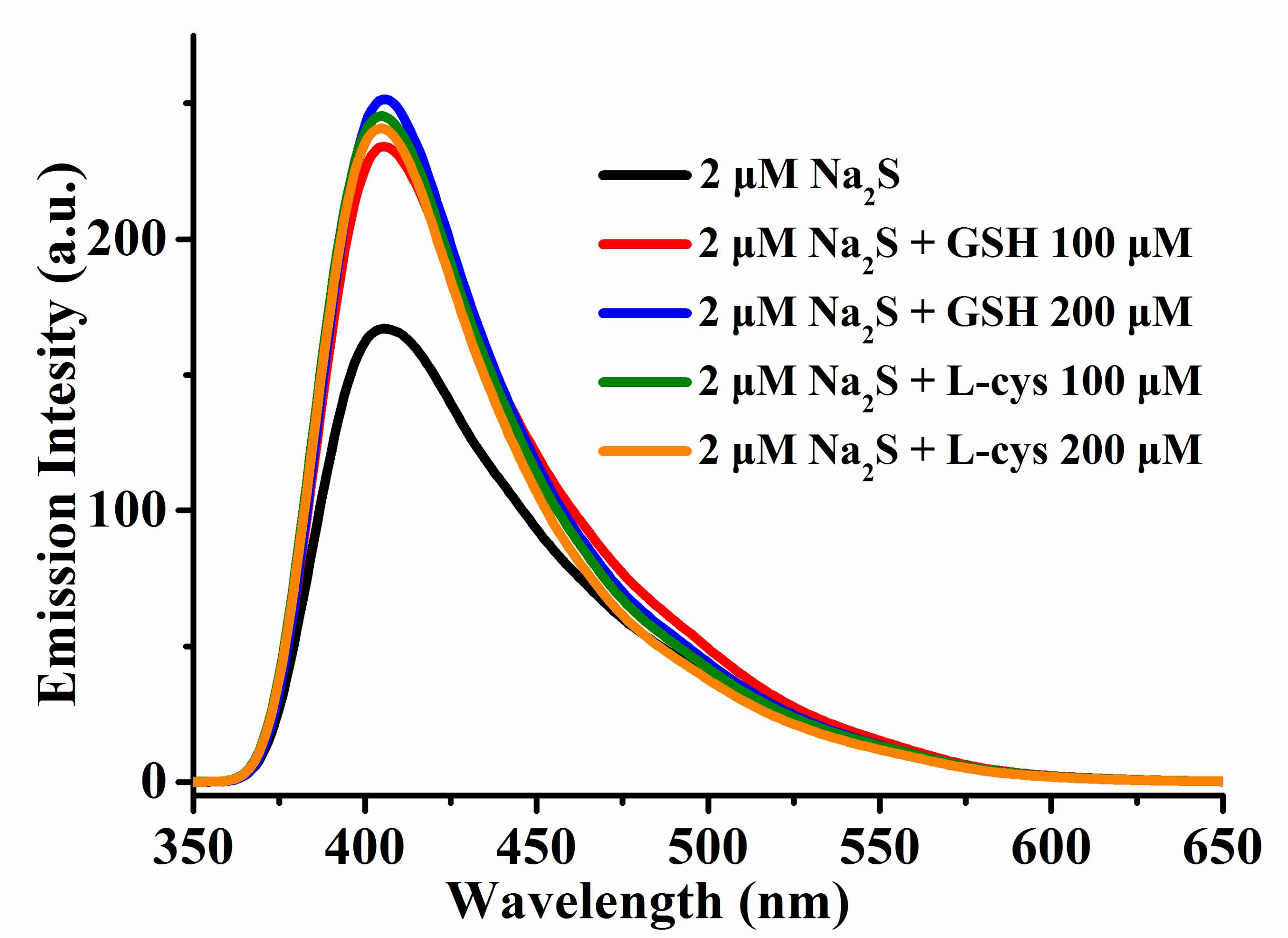


**Supplementary Figure S12 |** interference experiments.

1. **The fluorescent images of HeLa cells without being fixed.**


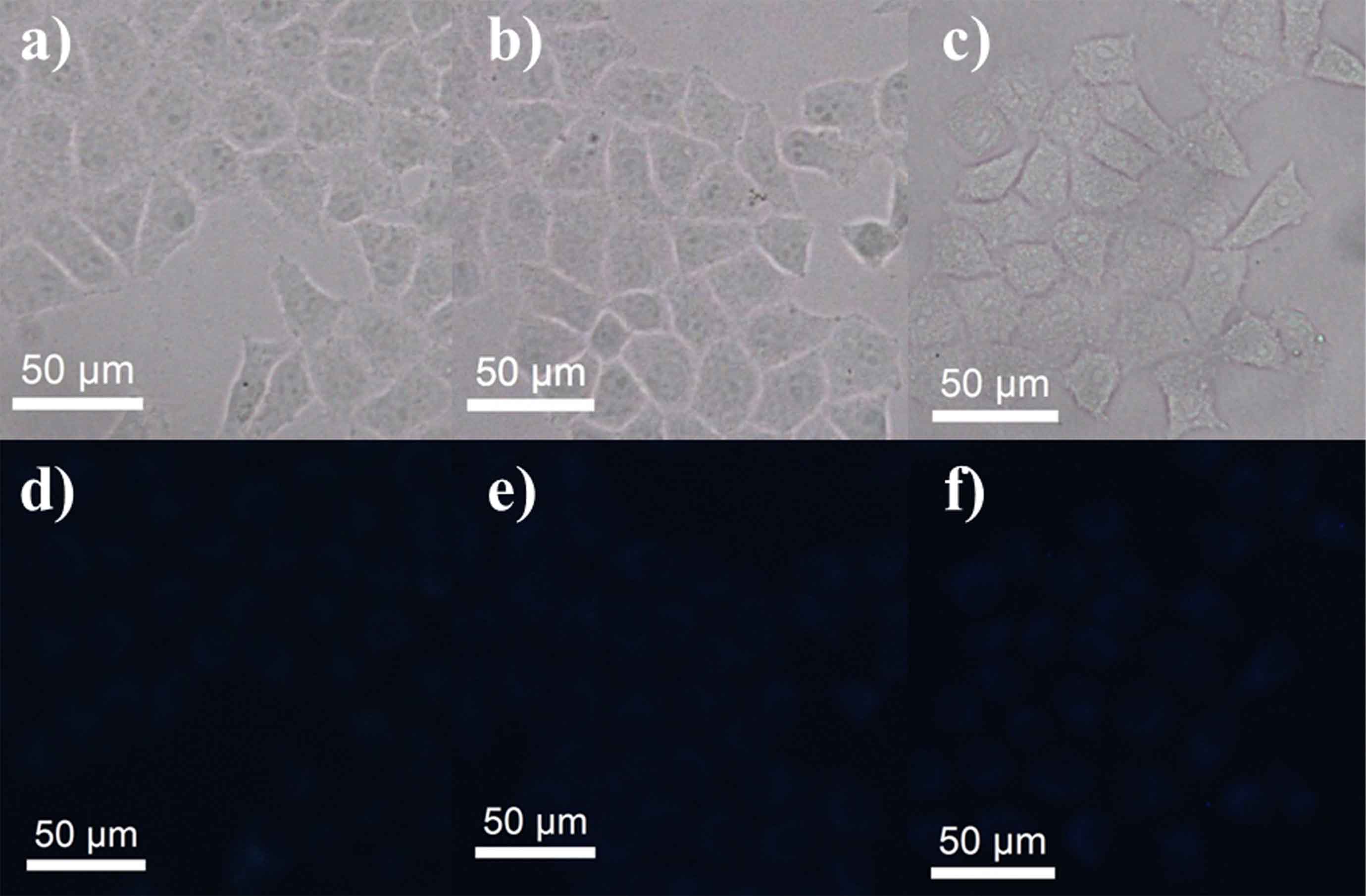


**Supplementary Figure S13 |** The fluorescent images of HeLa cells containing CD-MONT-2’ in PBS incubated with increasing concentrations of Na_2_S for 15 min at 37 °C: (d) 0 μM, (e) 1 μM, and (f) 50 μM; (a), (b), and (f) are the bright field images of (d), (e), and (f).
